# Variation in inhibitory control does not influence social rank, foraging efficiency, or risk taking, in red junglefowl females

**DOI:** 10.1007/s10071-022-01598-5

**Published:** 2022-02-04

**Authors:** Laura Clare Garnham, Robert Boddington, Hanne Løvlie

**Affiliations:** 1 Department of Physics, Chemistry and Biology, IFM Biology, Linköping University, 581 83 Linköping, Sweden; 2 School of Biological Sciences, University of Manchester, Manchester, M13 9PL UK

**Keywords:** Cognition, Foraging, Impulsivity, Inhibitory control, Risk taking, Social rank

## Abstract

Individual variation in cognition, seen in many taxa, is not well understood, despite its potential evolutionary consequences. Inhibitory control is an aspect of cognition which differs between individuals. However, how selection could act on this variation remains unclear. First, individual consistency over time of behaviours affected by inhibitory control, and how these behaviours relate to each other, is not well understood. Second, consequences in ecologically relevant contexts of variation in behaviours affected by  inhibitory control, are scarcely investigated. Therefore, we explored the temporal consistency and inter-relatedness of two behaviours influenced by inhibitory control (impulsive action and persistence) and how these link to social rank, foraging efficiency, and risk taking in adult female red junglefowl (*Gallus gallus*). We measured impulsive action in a detour test, and persistence in both a detour test and a foraging test. Impulsive action and persistence, measured in a detour test, were moderately consistent over time, and positively correlated. This implies that selection could act on inhibitory control via these behaviours, and selection on one behaviour could affect the other. However, we found no evidence of links between inhibitory control and social rank, foraging efficiency, or risk taking. This implies that selection may not act on inhibitory control via these measures, and that, in general, there may be a lack of strong selection on inhibitory control. This, in turn, could help explain individual variation in this aspect of cognition. Future research should explore the specificity of when inhibitory control has implications for individuals, and continue to investigate how variation in cognitive traits influences how individuals behave in contexts with potential evolutionary implications.

**Supplementary Information:**

The online version contains supplementary material available at 10.1007/s10071-022-01598-5.

## Introduction

Individual variation in cognition (i.e., how individuals acquire, process, store, and act on information from their environment, Shettleworth 2010) is often observed across taxa (e.g., Bensky and Bell 2018; Croston 2016; Koppik et al. 2015). Individual differences in cognition may result in individual differences in behaviour. Behaviours that differ consistently over time can be subject to selection (Smith and Blumstein 2008) and, thus, so can the aspects of cognition that affect these behaviours. An aspect of cognition may influence multiple behaviours. Thus, if these behaviours are correlated, selection on one of them could indirectly affect selection on the other interrelated behaviours. Furthermore, to determine how selection could acts on aspects of cognition, we need to explore how the behaviours affected by cognition link to individual outcomes in ecologically relevant contexts (e.g., Morand-Ferron and Quinn 2015; Thornton et al. 2014). Nevertheless, while there is an increasing interest in the consequences of individual variation in cognition (Ashton et al. 2018; Branch et al. 2019; Huebner et al. 2018; Isden 2013; Madden et al. 2018; Maille and Schradin 2016; Mery and Kawecki 2003; Miler et al. 2018; Minter et al. 2017; Raine and Chittka 2008; Shaw et al. 2019), these studies, typically, do not explore the temporal consistency of variation between individuals in, or correlations between, behaviours affected by the aspects of cognition they investigate (but see Ashton et al. 2018; Huebner et al. 2018; Minter et al. 2017; Shaw et al. 2019). Furthermore, the majority of the relatively few studies that investigate the consequences of cognitive variation tend to explore one potential consequence (but see Huebner et al. 2018; Shaw et al. 2019), thus obtaining a narrow picture of the potential consequences of cognitive variation.

Inhibitory control (a.k.a., impulse control, Coppens et al. 2010) is an aspect of cognition which typically varies between individuals of the same species (e.g., MacLean et al. 2014; Langley et al. 2020; Lucon-Xicatto et al. 2020a; Szabo et al. 2020). However, we do not know yet how inhibitory control is affected by selection. To begin with, little is known about temporal consistency in behaviours influenced by inhibitory control (but see, Kabadayi et al. 2018; Macario et al. 2020; Ryding et al. 2021; van Horik et al. 2018), nor how these behaviours correlate with each other. Behaviours influenced by inhibitory control include impulsive behaviours. For example, better inhibitory control links to lower levels of impulsive action (i.e., the inability to inhibit motor responses, Broos et al. 2012; Nautiyal et al. 2017) and persistence (i.e., continuing to use a previously adaptive response after it ceases to be adaptive, Evenden 1999) (Adinoff et al. 2007; Dalley et al. 2011; Devos et al. 2015; Schippers et al. 2017; Winstanley et al. 2006). Some studies find connections between these impulsive behaviours (e.g., Garner and Mason 2002), whereas others do not (e.g., Brucks et al. 2017; Johnson-Ulrich and Holekamp 2020; van Horik et al. 2018). Along with affecting impulsivity, inhibitory control can also influence how individuals make decisions (MacLean et al. 2014) and adapt to changing situations (Gilbert and Burgess, 2008; Lucon-Xicatto et al. 2017). Thus, variation in inhibitory control could be expected to affect individual outcomes in ways which could have evolutionary consequences. Evidence for this includes that better inhibitory control has been associated with more appropriate responses to changing social environments (e.g., Amici et al. 2008), a higher chance of attracting mates (e.g., Boogert et al. 2011), raising offspring to independence (e.g., Ashton et al. 2018; Minter et al. 2017), and higher social rank (e.g., Higley et al. 1992; Krakowski 2003) the latter of which can provide increased access to resources such as food or mating partners (Andersson 1994; Elwood and Arnott 2012). Overall, through investigating (i) temporal consistency in individual variation in behaviours that inhibitory control affects, (ii) correlations between these behaviours, and (iii) consequences of variation in these behaviours for individual outcomes in multiple ecologically relevant contexts, we can better understand how selection could act on inhibitory control. Despite this, there is currently a scarcity of studies which investigate any of these topics.

There are gaps in our knowledge of the consequences of variation in inhibitory control for individual outcomes. To begin with, while a link between social rank and inhibitory control has been detected (eg., Higley et al. 1992; Krakowski 2003), whether this link is due to inhibitory control playing a role in the establishment of social rank, or individuals with better inhibitory control being better at holding on to higher social rank, is unclear. That inhibitory control was not found to play a role in the establishment of social rank in red junglefowl, *Gallus gallus* (Garnham et al. 2019) suggests that the latter may explain a link between social rank and inhibitory control. However, further exploration is needed to confirm this. Further gaps in our knowledge come from that there are ecologically relevant contexts in which the potential consequences of individual variation in inhibitory control are scarcely explored. Two such contexts are foraging and responses to predators. In general, the effect of individual variation in inhibitory control (or even in other aspects of cognition) on foraging, or response to predators, lacks thorough investigation. In terms of foraging, individuals benefit from high foraging efficiency (i.e., obtaining more energy in less time, Emlen 1966; Mangel and Clarke 1986). Individuals with poorer inhibitory control could be more persistent when trying to acquire difficult to obtain food (as seen in van Horik et al. 2018). This could lead to lower foraging efficiency if other, easier to obtain, food sources are present, or alternatively, to higher foraging efficiency, if the food source is important and possible, but difficult to obtain. Regarding the relationship between inhibitory control and response to predators, inhibitory control could affect the risks that individuals take under the perceived threat of predation. Individuals that take more risks (i.e., are more active) under perceived threat of predation, increase their chances of obtaining resources, but also of being injured or killed (Dammhahn et al. 2018; Réale et al. 2010a; Stamps 2007). The link between inhibitory control and risk taking is, in general, unclear. In terms of risky decision making, humans and rats, *Rattus Norvegicus*, with poorer inhibitory control are more likely to choose riskier options that can have a higher payoff, but a lower chance of success, or a possibility of punishment (Freeman and Muraven 2010; Gabriel et al. 2019). In contrast to this, more proactive common waxbills, *Estrilda astrild* (i.e., those that are more risk taking in general, Réale et al. 2010b; Dammhahn et al. 2018), and bolder zebrafish, *Danio rerio*, and guppies, *Poecilia reticulata*, have been found to perform better in inhibitory control tests (Gomes et al. 2020; Lucon-Xiccato et al. 2020b). Overall, while individual variation in inhibitory control could have evolutionary implications by affecting individual outcomes in the ecologically relevant contexts of foraging and response to predators, this is not yet well explored.

We, therefore, here explored the following topics in female red junglefowl. First, whether behaviours shaped by inhibitory control, specifically impulsive action and persistence, showed individual consistency over time, tested by measuring these  behaviours twice in a detour test, three months apart. Second, whether we observed correlations between behaviours influenced by inhibitory control (namely impulsive action, persistence measured in a detour test, and persistence measured in a foraging test). Third, whether individual variation in behaviours shaped by inhibitory control linked to individual outcomes in terms of current social rank (investigated through same-sex staged contests), foraging efficiency (investigated with a foraging test), and risk taking (specifically under perceived predation threat, investigated with a simulated predator attack). Red junglefowl naturally form social hierarchies in which high ranking individuals produce more offspring (Collias et al. 1994), forage on food which can be patchily distributed and vary in ease of access (e.g., seeds, fruits, and invertebrates, Collias and Collias 1967), and are a prey species in their natural habitat (Evans et al. 1993; Schaller 1984Borah et al. 2009). This, along with that red junglefowl are increasingly used for behavioral and cognitive studies (e.g., Boddington et al. 2020, Rubene and Løvlie 2021, reviewed in Garnham and Løvlie 2018), made them ideal for this study.

## Methods

### Study population and housing

In November 2019 and February 2020, we used 30 sexually mature female junglefowl (36 weeks old in November and 50 weeks old in February). We only used females because adult males are not very willing to work for food rewards (Zidar et al. 2018) which was required in some of our tests. These females (hereafter referred to as ‘subjects’) came from a captive, pedigree bred, population maintained by Linköping University, Sweden (described in Sorato et al. 2018). All subjects were, as chicks, marked with unique numbered wing tags, enabling individual identification. The subjects used in this study differed slightly in early experiences due to a previous experiment (Garcia et al. in prep). Until they were five weeks old, these subjects had been raised at Linköping University. During this time, some subjects (n = 11) were raised in smaller groups (n_replicates_ = 4) , each consisting of seven individuals, whereas others (n = 19) were raised in larger groups (n_replicate__s_ = 3), each consisting of 16 individuals (more details in supplementary information). Twenty-five of the subjects used here experienced a battery of cognitive and personality tests during their first five weeks of life, in this earlier study (more than seven months prior to the current study), while five of them did not. At five weeks old, subjects were moved from Linköping University to an agricultural college (Vreta Gymnasiet) and housed in adult chicken facilities, where they were housed during this study. Specifically, during this study, all subjects were housed in an enclosure (6 m^3^) with sawdust for substrate and access to an outdoor area (250 × 260 × 400 cm; H x W x L) during the day time. We kept subjects on a 12:12 light cycle with lights on from 7am to 7 pm. While at adult facilities, all subjects lived in the same enclosure and were all exposed to the same husbandry practices. Therefore, from more than seven months prior to the current study, all subjects had similar experiences. Due to logistical constraints, our red junglefowl were housed with 11 white leghorn females from 5 weeks of age. As our junglefowl and the white leghorns had been together for over 30 weeks before testing commenced, their social group should have been stable at the time of testing. At the end of the study, all subjects continued to be maintained by Linköping University.

### General testing procedures

We conducted all testing between 9am and 6 pm and tested all subjects individually. Subjects took part in tests in this order: detour test, foraging test, simulated predator attack test, and staged contests (jn November 2019), and repeat testing in a detour test (in February 2020). For all tests, we collected data by direct observation. All tests were performed in the same way, and used the same equipment and set up for all subjects. Thus, there were no differences in how subjects experienced the tests that could have affected how they performed during testing. Further, no subjects experienced disturbances during testing. Testing was conducted outside of the subjects’ home pen, in familiar indoor rooms containing test arenas, which subjects were taken to by the observers, to prevent focal subjects being disturbed by other subjects in the home pen. To prevent observer bias, the subjects’ levels of impulsive action and persistence were not known to the observers in other tests. Food motivation can affect performance on cognitive tests (e.g., Rowe and Healy 2014; Smulders 2019; van Horik et al. 2018), thus we took steps to avoid variation in food motivation. First, we provided subjects with ad libitum food in their home pen, so none were food deprived at the time of testing. Second, if subjects repeatedly showed low food motivation in any test, we removed them from further testing and from our analyses. In total, this happened for four subjects in November (in the original inhibitory control tests) and one subject in February (in the repeat inhibitory control tests).

### Detour test

We used a detour test (Boogert et al. 2011; MacLean et al. 2014) to measure impulsive action and persistence, therefore, assessing inhibitory control. We conducted pre-training for the detour test, and the test itself, in an arena (82 × 53 × 50 cm; L x W x H). Before testing, we trained subjects (n = 30) to use a detour (i.e., to insert their head into the center of an opaque tube, 15 L × 7.5 Ø cm, from the side, to obtain a reward, one mealworm, sensu MacLean et al. 2014; Ryding et al. 2021). We initially helped subjects to learn this detour by tapping on the opaque tube, or guiding them to the reward in the tube, using extra rewards. The training criteria subjects had to reach before we assumed they had learnt the detour, was the retrieval of the reward from the tube five consecutive times without any help. It was easy for observers to notice when a subject had reached the training criteria, as doing so involved the subject performing five consecutive, specific, easily recognisable actions (sensu Garnham et al. 2019; Ryding et al. 2021). There was little variation in the number of training trials subjects needed to learn the detour, and all (bar one subject) learnt this relatively fast (most within 10 presentations of the opaque tube). Thus, we do not believe that variation in exposure to detour training influenced performance in the detour test. Further, as all but one subject (same as mentioned earlier) reached the training criterion quickly, this training did not appear difficult for our subjects. The one subject that did not learn the detour within three training sessions did not appear to struggle with the test but rather was not interested in obtaining the reward due to low food motivation. This subject was excluded from further testing.

Immediately after a subject reached our training criteria, she began the detour test. In total, 29 subjects participated in this test. We began each trial of this test by placing the subject and a transparent tube (15 L × 7.5 Ø cm) with a reward inside, at opposite short sides of the arena with the long side of the tube facing the subject (sensu Ryding et al. 2021). All subjects, but five, had already experienced transparent objects prior to this detour test due to participating in detour tests as chicks, more than seven months earlier (Garcia et al. in prep). As experience with transparent objects can affect performance in the detour test (van Horik et al. 2018), we took this difference in previous experience into account in our analyses, by conducting analyses on data both with, and without, these subjects included. In our detour test, each subject had to use the previously learned detour to access the reward without being helped. We gave each subject five trials in succession, and up to one minute per trial. Five trials captured variation among our subjects, while reducing the effect of learning over successive trails (as seen in Kabadayi et al. 2018; Ryding et al. 2021; van Horik et al. 2018). Similar to previous studies (Boogert et al. 2011; Garnham et al. 2019; MacLean et al. 2014; Ryding et al. 2021), we measured impulsive action as the number of these five trials in which a subject attempted to reach the reward directly by pecking at the transparent tube (we called this measure ‘Impulsive action’, and a higher score implied higher impulsive action). We measured persistence as the subject’s total number of pecks at the transparent tube across these five trials (termed ‘Persistence DT’, where DT referred to detour test, and a higher score implied higher persistence). Higher impulsive action and higher persistence imply lower inhibitory control (Adinoff et al. 2007; Dalley et al. 2011; Devos et al. 2014; Schippers et al. 2017; Winstanley et al. 2006). We considered subjects that did not approach the tube within one minute in three consecutive trials to have failed the detour test. Three subjects failed the test, again due to low food motivation, and were excluded from further testing. Two of these subjects had not experienced the detour test as chicks. Therefore only three subjects (of five) that had not experienced the detour test as chicks went on to be included in further testing and analyses.

We assessed the food motivation of all subjects that completed the detour test (n = 26), by seeing how many mealworms, out of five (presented sequentially), they would eat. All the subjects that completed the detour test showed high food motivation (all, but three subjects, ate all five mealworms and those three that did not eat five mealworms ate four mealworms). Thus, we believe that there were no significant differences in food motivation between subjects that completed the detour test and thus that food motivation did not influence the inhibitory control measures used in our analyses.

To determine if our measures of ‘Impulsive action’ and ‘Persistence DT’ were consistent within subjects over time, we repeated the detour test three months later (in February 2020), without any new training beforehand. One subject, which passed the detour test the first time, failed it this time. Therefore, the temporal consistency of impulsive action and persistence, two behaviours influenced by inhibitory control, was tested in 25 subjects.

### Staged contests

To investigate whether inhibitory control was linked to a subject’s relative social rank in a group, we staged pairwise contests (sensu Garnham et al. 2019), in which each contesting pair contained one subject with a relatively higher impulsive action score (i.e., 4–5) and one with a relatively lower impulsive action score (i.e., 0–2). As our subjects were already part of a social group, by staging these contests we investigated links between current social rank and inhibitory control, not whether inhibitory control affected the establishment of social rank (as investigated in Garnham et al. 2019). We held contests in a room familiar to our subjects (used for the foraging test and simulated predator attack which were conducted prior to this test) with sawdust as substrate. A piece of apple was provided to give the subjects something to compete over and thus increase our chances of observing agonistic behaviours. To reduce the effect of morphology on contest outcome, we chose opponents that had comb (mm), tarsus (mm) and weight (g) within 10% of each other (sensu Favati et al. 2014; Garnham et al. 2019). We measured comb and tarsus with digital callipers, and weight using an electronic scale, prior to the detour test. Morphologically matched opponents could not be found for seven subjects, so they were excluded from the contests, resulting in 21 pairwise contests between 19 subjects. Most subjects participated in two contests, two subjects participated in three contests and five subjects participated in only one contest. This came from the need to have data from enough contests for our analyses while having as few contests as possible for ethical reasons. We considered a subject to have a higher social rank if her opponent avoided her three consecutive times (sensu Garnham et al. 2019) and contests were gently terminated immediately after this occurred. We only tested each pair once, as, in red junglefowl, individual rank is generally stable over time (Collias 1943; Collias and Collias 1996). Furthermore, during the time period over which data was collected, the subjects’ social group did not undergo any changes that could have influenced individual rank (e.g., removal, or addition, of birds).

### Foraging test

To measure how inhibitory control linked to foraging efficiency, subjects that had completed the detour test (n = 26) took part in a foraging test in a room (284 × 298 × 305 cm; L x W x H) in which they were presented with nine glass petri dishes with lids (84.5 Ø mm) equally spaced 80 cm from each other. Each petri dish had three accessible mealworms on the top of, and three inaccessible mealworms below, its lid. Therefore, we created a foraging situation in which remaining at a dish trying to access inaccessible food, rather than leaving it to find accessible food elsewhere, led to lower foraging efficiency. Due to potential individual foraging speed differences, and wanting to limit how long subjects spent in testing, the test had three possible end points: 1) As subjects could potentially visit all patches in two minutes (confirmed by pilot tests), if a subject spent more than two minutes in the test, the test ended when she had visited three patches, as this was enough to get sufficient data on her foraging efficiency. 2) If a subject had visited more than three patches, but less than seven patches, by two minutes, the test ended at two minutes. 3) If a subject had visited seven patches before two minutes, the test ended at the seventh patch, as we needed to leave at least a couple of patches un-foraged for the simulated predator attack (described below). To assess a subject’s foraging efficiency, we recorded the number of patches she visited, divided by the time she spent foraging (i.e., eating, pecking at food, scratching for food, or walking directly to a food patch). A higher score implied higher foraging efficiency. Subjects always ate all the accessible worms at a patch before moving to another patch, thus no subjects obtained a higher foraging efficiency by leaving accessible worms unconsumed. To test if persistence in this test could affect foraging efficiency, and if individuals showed similar levels of inhibitory control in both the detour and foraging tests (sensu van Horik et al. 2018), we recorded persistence in this test as how many times a subject pecked towards the inaccessible food for all patches (‘Persistence FT’, FT referred to foraging test, a higher score implied higher persistence). As all subjects ate available worms when they came across them and tried to reach the inaccessible worms, we feel that all subjects had similar levels of food motivation during this test.

### Simulated predator attack test

Immediately after the foraging test, to explore whether inhibitory control was linked to risk taking under perceived threat of predation, subjects (n = 26) were exposed to a simulated predator attack test (sensu Favati et al. 2016). We released a black model outline of a hawk (62 × 31 cm; W x L) down a zip-line placed diagonally across the room in which the foraging test had taken place (from 287—114 cm above ground level). The model imitated the flying silhouette of a hawk of the Accipiter family. Hawks and other raptors are natural predators of *Gallus* species (Evans et al. 1993; Collias and Collias 1967). Thus, this outline was predicted to trigger anti-predator behaviour in our subjects (e.g., Evans et al. 1993; Wilson and Evans 2009). We scored the intensity of each subject’s response to the model, which could indicate how threatening they perceived the simulated attack as being, thus we termed this measure ‘Threat perception’ (0–5, 5 suggests that the subject found the predator model very threatening, Table 1). As a subject may remain vigilant for longer, and, therefore, take longer to return to foraging after being exposed to a predator stimulus, if they perceive it as a greater threat, we also measured latency to resume foraging and termed this measure ‘Time spent vigilant’. ‘Threat perception’ reflected a subjects initial response to the simulated predator, whereas ‘Time spent vigilant’ reflected a more delayed response. In this test, an a subject that perceived a greater level of threat (i.e., had higher ‘Threat perception’) can be considered more risk taking than one that perceived a lower level of threat, but took the same amount of time to return to foraging (i.e., same ‘Time spent vigilant’). Therefore, we made a combined ‘Risk taking’ measure (‘Time spent vigilant’/’Threat perception’), which we used in our analyses, in which a lower value indicated higher risk-taking. All but three of the subjects responded to the predator stimulus by running and/or alarm calling (typical responses to predators in fowl, Evans et al. 1993; Wilson and Evans 2009). Thus, overall, our study subjects appeared to perceive the predator stimulus as a threat.

**Table 1:** Scoring behavioural responses of female red junglefowl to a simulated avian predator attack

Score	Description of behavioural response
0	Shows no behavioural response
1	Takes a few quick steps to move out of line of the predator model, but otherwise shows no further response
2	Either runs to edge of room then and stops, or alarm calls for less than five seconds
3	Either tries to escape, or alarm calls for more than five sec
4	Both tries to escape, and alarm calls, for any length of time
5	Flies around in the room and alarm calls for any length of time

How subjects responded to a simulated predator attack by a model hawk was scored on a scale on 0–5, a higher score indicated a more intense response. The scale was developed from Favati et al. (2016)

### Welfare statement

All the subjects used in this study had been regularly handled since soon after hatching, were accustomed to human presence, and were habituated to being alone in testing arenas prior to testing. When in their home pen, all subjects had access to enrichment (perches, shelters, and sawdust for dustbathing) and ad libitum access to commercial poultry food and water, thus they were never starved prior to testing. All training and testing sessions lasted max 15 min, and we returned subjects to their home pen for at least one hour rest between sessions. Subjects were also given at least one hour rest between contests. Regarding our simulated predator test, we accept that the model predator acted as a stressor to our subjects. However, the stress they experienced was short lived, and all bar two subjects returned to foraging within two minutes after the simulated attack (the two that took longer to return to foraging did so within five minutes). Thus, we do not think the experience of the simulated predator attack had any long-term detrimental effects on our subjects. Concerning our social rank tests, previous pilot work in our group has found that using mirrors does not produce relevant behavioural data. Further, as chicken have a higher flicker fusion frequency than humans (Lisney et al 2011), using video playback would have been unlikely to produce relevant behavioural data as our subjects would most likely have perceived this as not normal behaviour. Consequently, contests with live individuals were necessary to investigate current social rank. We arranged contest pairs, and only had one contest per pair, to make the overall number of contests as low as possible. We were always ready to break up aggressive interactions if they occurred, and veterinary care was readily available if needed. No contests resulted in any serious fighting and no subjects obtained any injuries.

### Statistical analyses

All analyses were conducted in R studio (v. 3.5.2). We used non-parametric statistical analyses, as our data did not meet the assumptions of parametric statistics. Results of statistical analysis with a p-value < 0.05 were considered significant.

For the first stage of our analyses, we investigated if how subjects were housed, or the replicate they were housed in, as chicks, biased our results. As this investigation was not a focus of this study, how it was conducted is described in the supplementary information. If we found an effect of how subjects had been housed, or the replicate they were housed in, as chicks, on any of our measures, for these measures we performed separate analyses on data from subjects housed in different ways, or in different replicates, as chicks.

To explore the temporal consistency of ‘Impulsive action’ and ‘Persistence DT', between November 2019 and February 2020, and relationships between ‘Impulsive action’, ‘Persistence DT’ and ‘Persistence FT’, measured in November 2019, we used Spearman’s rank correlations. Spearman’s rank correlations were also used to investigate whether ‘Impulsive action’, ‘Persistence DT’ or ‘Persistence FT’, measured in November 2019, linked to ‘Foraging efficiency’, or ‘Risk taking’. To examine whether ‘Impulsive action’, ‘Persistence DT’ or ‘Persistence FT’, measured in November 2019, predicted social rank measured in same-sex pairwise contests, we used paired Wilcoxon signed-rank tests (winners vs. losers). While higher ‘Impulsive action’, ‘Persistence DT’, and ‘Persistence FT’ all reflected lower inhibitory control, we analysed each of these behaviours separately as each may be affected by different aspects of inhibitory control.

As previous experience could affect performance in cognitive tests (e.g., Boogert et al. 2018; Dochtermann 2010), we ran all our analyses both with, and without, the three subjects that did not experience the detour test as chicks. Results are reported from the analyses with all subjects, and any qualitative differences in results that came from removing the subjects that did not experience the detour test as chicks, are given for each result below. We also ran the analyses with, and without outliers. First, we only removed extreme outliers (i.e., points more than three times the interquartile range from upper and lower quartiles, Hodge and Austin 2004; Levend Asikoglu 2017), then we removed both extreme and mild outliers (defined as points between 1.5 and three times the interquartile range from upper or lower quartiles, Hodge and Austin 2004; Levend Asikoglu 2017). However, neither of these methods of outlier removal qualitatively affected our results, thus we only report results including all data.

## Results

### Overview of effects of housing as chicks on test performance as adults

In terms of behaviours affected by inhibitory control, how subjects were housed as chicks did not affect ‘Impulsive action’ or ‘Persistence DT’, but did affect ‘Persistence FT’. Regarding our other behavioural measures, only ‘Risk taking’ in the simulated predator test was affected by how subjects were housed as chicks. Finally, how subjects were housed as chicks did not affect how likely they were to win contests in the current study. Statistics along with a more detailed description of these effects can be found in the supplementary information. Due to these findings, for analyses involving ‘Persistence FT’ or ‘Risk taking’, subjects that had been housed in smaller, or in larger groups, as chicks, were considered separately. For all other analyses, all data were analysed together. We only found one significant pairwise comparison between replicates (see supplementary information for details), thus replicate was not considered in further analyses.

### Temporal consistency of behaviours influenced by inhibitory control

**Fig. 1:**
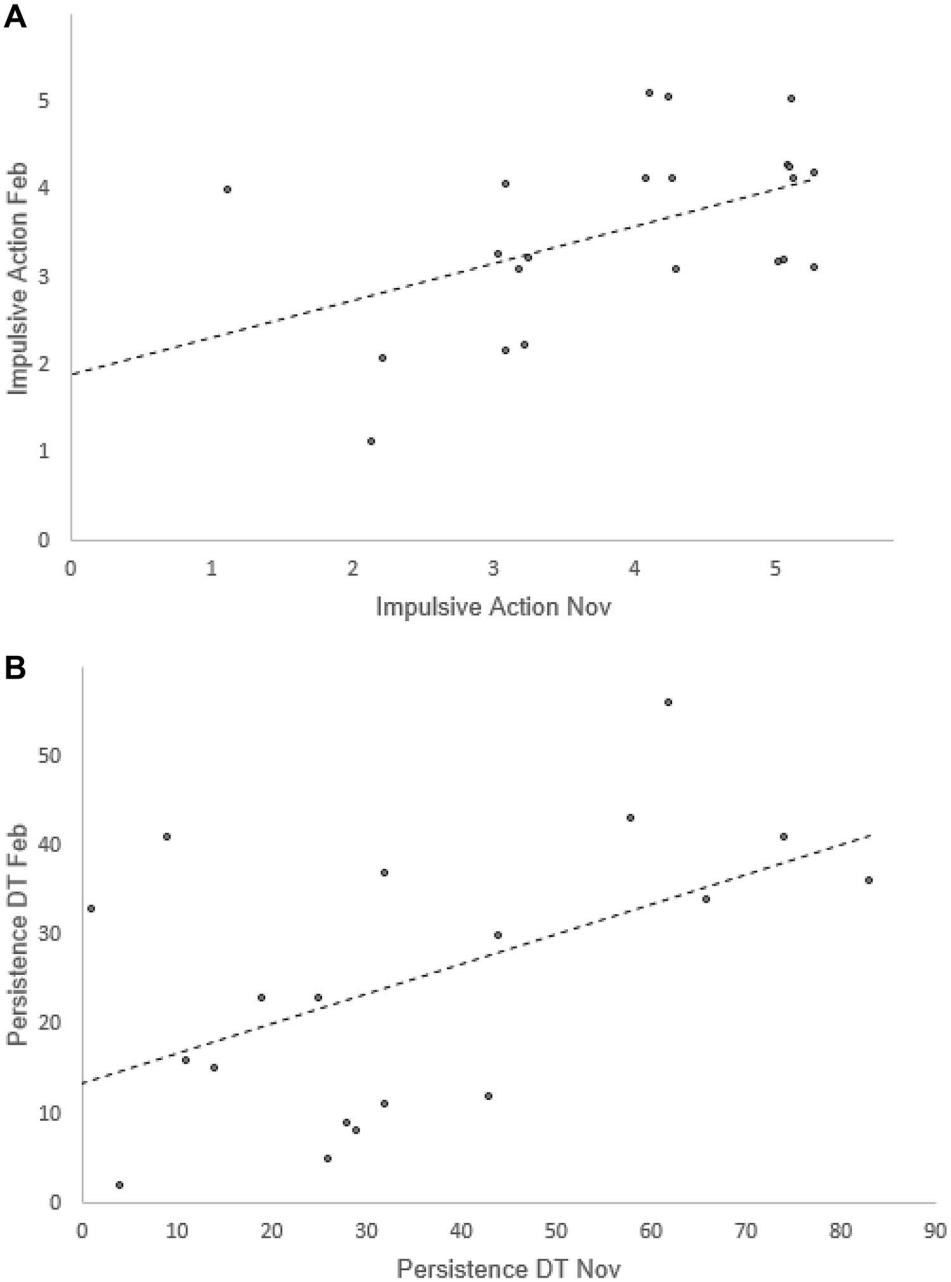
Consistency over three months in impulsive action and persistence (measured in a detour task), in red junglefowl females. ‘Impulsive action’ = the number of trials, out of five, in which a female pecked at a transparent tube containing a reward in a detour task. ‘Persistence DT’ = a female’s total number of pecks at a transparent tube containing a reward across all five trials in a detour task. **A** Consistency in ‘Impulsive action’ between November 2019 (‘Impulsive Action Nov’) and  February 2020 (‘Impulsive Action Feb’). Data is jittered to show all data points. **B** Consistency in ‘Persistence DT’ between November 2019 (‘Persistence DT Nov’) and February 2020 (‘Persistence DT Feb’). Only data from females that took part in cognitive testing as chicks (for both tests *n* = 22) are shown (see main text for details). Each point on the graphs represents a female. The figure was made in Excel

The two ‘Impulsive action’ measures taken three months apart showed moderately high consistency (n = 25, r_s_ = 0.41, p = 0.04, Fig. 1A). The two measures of ‘Persistence DT’ also showed moderately high consistency, although this was non-significant when including all subjects (n = 25, r_s_ = 0.38, p = 0.06). Removing the three subjects which did not experience the detour test, as chicks, caused a qualitative change in the relationship between our two measures of ‘Persistence DT’ in that this relationship became significant (n = 22, r_s_ = 0.45, p = 0.03, Fig. 1B).

### Correlations between behaviours influenced by inhibitory control

We found a strong, positive correlation between ‘Impulsive action’ and ‘Persistence DT’ (n = 26, r_s_ = 0.70, p < 0.01), but neither were correlated significantly with ‘Persistence FT’ in subjects that were housed in smaller, or larger, groups as chicks (subjects housed in smaller groups as chicks: ‘Impulsive action’: n = 10, r_s_ = 0.45, p = 0.19; ‘Persistence DT’: n = 10, r_s_ =  0.39, p = 0.26; subjects housed in larger groups as chicks: ‘Impulsive action’: n = 16, r_s_ = 0.10, p = 0.69; ‘Persistence DT’: n = 16, r_s_ = 0.03, p = 0.92).

### Links between behaviours influenced by inhibitory control and social rank

We found no connections between ‘Impulsive action’, ‘Persistence DT’, nor ‘Persistence FT’ and social rank measured in same-sex pairwise contests (‘Impulsive action’: mean ± SE, contest winners: 3.48 ± 0.27, contest losers: 3.95 ± 0.21, V = 143.00, p = 0.15, 95% confidence interval = 4.61 × 10^–5^, 2.00; ‘Persistence DT’: mean ± SE, contest winners: 28.11 ± 3.77, contest losers: 37.44 ± 4.40, V = 158.50, p = 0.14, 95% confidence interval = -3.50, 25.00; ‘Persistence FT’: mean ± SE, contest winners: 9.26 ± 0.65, contest losers: 11.93 ± 1.53, V = 151.50, p = 0.22, 95% confidence interval = -1.03, 4.65; for all: n = 19). There was insufficient data to separately look at the effect of ‘Persistence FT’ on contest outcome in subjects housed in different ways as chicks.

### Links between behaviours influenced by inhibitory control and foraging efficiency

We found no links between ‘Impulsive action’, ‘Persistence DT’, nor ‘Persistence FT’ and ‘Foraging efficiency’ (‘Impulsive action’: n = 26, r_s_ = 0.16, p = 0.43; ‘Persistence DT’: n = 26, r_s_ = 0.09, p = 0.66; ‘Persistence FT’: subjects housed in smaller groups as chicks: n = 10, r_s_ = -0.13, p = 0.71; subjects housed in larger groups as chicks: n = 16, r_s_ = -0.23, p = 0.36).

### Links between behaviours influenced by inhibitory control and response to a simulated predator

We found no correlations between ‘Impulsive action’, ‘Persistence DT’, nor ‘Persistence FT’ and ‘Risk taking’ (‘Impulsive action’: subjects housed in smaller groups as chicks: n = 10, r_s_ = -0.32, p = 0.37; subjects housed in larger groups as chicks: n = 16, r_s_ = 0.27 p = 0.28; ‘Persistence DT’: subjects housed in smaller groups as chicks: n = 10, r_s_ = 0.36, p = 0.31; subjects housed in larger groups as chicks: n = 16, r_s_ = -0.1, p = 0.70; ‘Persistence FT’: subjects housed in smaller groups as chicks: n = 10, r_s_ < 0.01 p = 0.1; subjects housed in larger groups as chicks: n = 16, r_s_ = -0.38, p = 0.13). Furthermore, the measures that we combined to make our ‘Risk taking’ measure (‘Threat perception’ and ‘Time spent vigilant’ were not linked to any our inhibitory control measures, when analysed separately: -0.3 < r_s_ < 0.05, p > 0.1).

## Discussion

Individual differences in cognition can result in individual differences in behaviour. To better understand how selection could act on these behaviours, and thus the aspects of cognition that affect them, it helps to know certain aspects. First, whether these behaviours differ consistently over time. Second, how these behaviours correlate with each other. Third, the consequences for individuals of variation in these behaviours. We here explored this in adult red junglefowl females, in terms of impulsive action and persistence, behaviours that are influenced by an aspect of cognition: inhibitory control (Adinoff et al. 2007; Dalley et al. 2011; Devos et al. 2014; Schippers et al. 2017; Winstanley et al. 2006). We found that impulsive action and persistence measured in a detour task were moderately consistent over time and positively correlated, whereas measures of behaviours influenced by inhibitory control taken in different tests did not correlate. We found no evidence of variation in inhibitory control being linked to individual outcomes in terms of social rank, foraging efficiency, or risk taking, all of which are ecologically relevant contexts in which individual outcomes could result in selection.

We here measured how consistent subjects were in inhibitory control and persistence, in a detour task, over a considerably long time (three months), which, to our knowledge, had not been explored previously. We show these measures to be moderately consistent over this time. Nevertheless, not all studies find consistency in behaviours affected by inhibitory control. For example, studies which investigate consistency in how inhibitory control affects performance across tasks tend to find no consistency, potentially because these tasks measure different aspects of inhibitory control (reviewed in Macario et al. 2020). Consistency in inhibitory control in other studies may also be affected by learning, with repeated experience of a task individuals have been found to demonstrate better inhibitory control (e.g., Kabadayi et al. 2018; Ryding et al. 2021; van Horik et al. 2018), or length of time between experiences of the task. That learning and memory can affect consistency in inhibitory control implies that individuals are flexible in how impulsively they behave and can improve and adapt to show levels of inhibitory control more appropriate to their current situation. In turn, this ability to be flexible could reduce consequences of variation in inhibitory control. Overall, consistency in inhibitory control, and what determines whether or not individuals show this, warrants further investigation. We observed a correlation between impulsive action and persistence measured in a detour test. This correlation may be due to both measures being taken from the same test (sensu Garner and Mason 2002). An alternative explanation is that both are measures of the same trait. That said, impulsive action and persistence appear to have different underlying gene expression (Ryding et al. 2021) and thus may be shaped by different aspects of inhibitory control. We found no correlation between inhibitory control measures (impulsive action and persistence) taken from a detour test and an inhibitory control measure (persistence) taken from a foraging test. This supports previous findings in which inhibitory control measures did not correlate across tests (dogs, *Canis Familiaris*: Brucks et al. 2017; spotted hyena, *Crocuta crocuta*: Johnson-Ulrich and Holekamp 2020), but contrasts a positive correlation between inhibitory control in a detour test and inhibitory control in a foraging test, in pheasant chicks, *Phasianus colchicus* (van Horik et al. 2018). Together, this suggests that relationships between different behaviours affected by inhibitory control may differ between species, or developmental stages, as the study on pheasants (van Horik et al. 2018) used chicks whereas we here used adult female red junglefowl. In terms of the latter, this could potentially be due to younger animals having poorer inhibitory control compared to adults, as individuals can learn to improve their inhibitory control (Kabadayi et al. 2018; Ryding et al. 2021; van Horik et al. 2018).

Unlike previous studies (e.g., Higley et al. 1992; Johnson-Ulrich and Holekamp 2020; Krakowski 2003), we found no connection between behaviours shaped by inhibitory control and current social rank, thus indicating a lack of connection between inhibitory control and social rank in red junglefowl females. Along with our previous finding that impulsive action did not influence the establishment of social rank in red junglefowl males or females (Garnham et al. 2019), this implies that, in general, inhibitory control may not influence social rank in this species. Poor inhibitory control has been linked to high levels of unrestrained and excessive aggression in rhesus macaques, *Macaca mulatta*, and vervet monkeys, *Chlorocebus pygerythrus* (Higley et al. 1992; Krakowski 2003). These studies (Higley et al. 1992; Krakowski 2003) imply that individuals with poorer inhibitory control are more likely to initiate fights against opponents they are unlikely to beat. This can lead to more impulsive individuals suffering more injuries and, because injured individuals are less able to defend their social rank, can result in them decreasing in rank. Aggression is well documented to show individual variation in females from *Gallus* species, both in the ancestor species (red jungefowl: Garnham et al. 2019; Kim and Zuk 2000) and its domestic descendants (domestic chickens: Bshary and Lamprecht 1994; Vallortigara 1992). Nevertheless, we did not observe high aggression in our subjects, which could explain why we did not see a link between inhibitory control and social rank here. Further, in spotted hyenas, social group size was shown to affect the observed link between inhibitory control and social rank (i.e., this link only appeared in larger social groups, Johnson-Ulrich and Holekamp 2020). While the society of spotted hyena differs considerably from that of red junglefowl, this finding may imply that social group size could also affect relationships between inhibitory control and social rank in other species, including red junglefowl. The social group we used here consisted of 30 individuals (41 if the white leghorns they were housed with were also included), which is larger than the upper limits of red junglefowl group sizes observed in the wild (ca 15 individuals, McBride et al. 1969, Sullivan 1991, Collias and Collias 1996). Thus, even if we would find a link between inhibitory control and social rank in larger groups than studied here, we do not think this would be ecologically relevant.

We also investigated whether behaviours influenced by inhibitory control connected with foraging efficiency and risk taking (the latter under perceived predation threat). Overall, the effect of variation in aspects of cognition on foraging efficiency and risk taking has been rarely investigated. Here, our results evidenced no connections between inhibitory control and foraging efficiency or risk taking. Regarding foraging efficiency, while we set up the foraging test so that individuals with poorer inhibitory control should have had poorer foraging efficiency, we did not find this connection. Nevertheless, while we did not find inhibitory control to affect foraging, in terms of how many patches of food a subject processed in a given time, inhibitory control still has the potential to affect other aspects of foraging. For example, a recent study in great tits, *Parus major*, found that birds with better inhibitory control were more able to switch to an alternative food source when this had high value (Coomes et al. 2021). In terms of risk taking, links to inhibitory control are still unclear with both positive (e.g., Gomes et al. 2020; Lucon-Xiccati et al. 2020b) and negative (e.g., Freeman and Muraven 2010; Gabriel et al. 2019) and now a lack of connections being found (this study). Overall, there is considerable scope for investigating how different aspects of inhibitory control affect different aspects of foraging and risk taking, thus improving our understanding of the effects of variation in cognition for individual outcomes in ecologically relevant contexts. Sex is another factor which could potentially affect how different behaviours affected by inhibitory control relate to each other, or how variation in inhibitory control affects individual outcomes. We here used only females and so could not explore sex differences. However, sex differences in inhibitory control have been demonstrated in other species (e.g., in humans: Thakkar et al. 2014; Weafer 2020; rats: Bayless and Daniel 2015; Nile tilapia, *Oreochromis niloticus*: Brandão et al. 2019; Guinea baboons, *Papio papio*: Lacreuse 2016; pheasants: Meier et al. 2017). Therefore, future research should aim to include both sexes when investigating the consequences of individual variation in cognition. In general, how inhibitory control connects to individual outcomes regarding social rank, foraging efficiency or risk taking may differ depending on species, context, or sex in focus.

That variation in inhibitory control may sometimes have positive outcomes (e.g., Amici et al. 2008; Higley et al. 1992; Krakowski 2003; Boogert et al. 2011; Ashton et al. 2018; Minter et al. 2017), sometimes no effect on outcomes (e.g., this study; Garnham et al. 2019), and sometimes negative outcomes for individuals (e.g., if higher impulsivity could potentially increase the likelihood of acquiring, or accessing difficult to obtain, resources), indicates that overall directional selection favouring better inhibitory control may be lacking. This could help explain why individual variation in inhibitory control is repeatedly observed within species across taxa (e.g., Langley et al. 2020; Lucon-Xicatto et al. 2020a; MacLean et al. 2014; Szabo et al. 2020). Studies investigating the consequences of individual variation in other aspects of cognition have also found better cognitive abilities to result in both positive (e.g., Ashton et al. 2018; Branch et al. 2019; Maille and Schradin 2016; Minter et al. 2017; Raine and Chittka 2008; Shaw et al. 2019), negative (e.g., Madden et al. 2018; Mery and Kawecki 2003; Miler et al. 2018) and no effect on outcomes for individuals in ecologically relevant contexts (e.g., Huebner et al. 2018; Isden 2013). Consequently, a lack of strong directional selection could partly explain why many species show individual variation in cognition in general.

In conclusion, in our red junglefowl females, we found moderately consistent temporal variation in inhibitory control (i.e., variation that selection could act upon) over a relatively long time (ca three months). However, this variation in inhibitory control was not linked to individual outcomes in the three ecologically relevant contexts we explored: social rank, foraging, or risk taking under the perceived threat of predation, contexts in which outcomes could have implications for the individual. Together with results from previous studies, our findings indicate that how different behaviours shaped by inhibitory control relate to each other, or influence individual outcomes with potentially evolutionary implications, may depend on factors such as species, developmental stage, context, or sex. To explore the generality and robustness of our findings, future studies are encouraged to include multiple populations of the same species and/or multiple species, to obtain a broader picture of the outcomes, for individuals, of cognitive variation. In addition, studies using domesticated animals could focus on multiple breed strains to broaden relevance to a wider audience (e.g., production, welfare). Overall, future research into how variation in cognition affects individual outcomes could explore the specificity of when such relationships are observed and when not. Further, that both positive, negative and a lack of effect on outcomes for individuals with better inhibitory control have been found across studies suggests that strong directional selection for better inhibitory control may be lacking, which could potentially help explain the maintenance of variation in inhibitory control.

## Supplementary Information

Below is the link to the electronic supplementary material.

Supplementary file1 (DOCX 16 KB)

## Data Availability

The datasets generated and analysed during the current study, as well as the code used for analyses, are available from the corresponding author on request.
