# Supplementary material for: Variation in inhibitory control does not influence social rank, foraging efficiency, or risk taking, in red junglefowl females
Source: Anim Cogn. 2022 Feb 4;25(4):867–79. doi: 10.1007/s10071-022-01598-5 (PMC9334373; doi:10.1007/s10071-022-01598-5)
Supplement: Supplementary file 1 — Supplementary file1 (DOCX 16 KB) [file 10071_2022_1598_MOESM1_ESM.docx]

**Supplementary information for 'Garnham, Boddington and Løvlie ‘Variation in inhibitory control does not influence social rank, foraging efficiency, or risk taking, in red junglefowl females’.**

**Animal cognition**

Laura C. Garnham^1^, Robert Boddington^1,2,^ Hanne Løvlie^1^

^1^Department of Physics, Chemistry and Biology, IFM Biology, Linköping University,

SE-581 83 Linköping, Sweden.

^2^School of Biological Sciences, University of Manchester, M13 9PL Manchester, UK.

Corresponding author: [laura.garnham@liu.se](mailto:laura.garnham@liu.se), +46766012705

**Investigating if housing, or replicate housed in, as chicks affected test performance in adults**

Background

The adult female junglefowl ('subjects') used in the study discussed in this manuscript differed slightly in early experiences due to a previous study (Garcia et al. in prep) that took place less than seven months before this study, when the subjects were chicks (from 0-5 weeks old). During this previous study, some subjects (n = 11) were housed in smaller groups (each consisting of seven individuals, n replicates = 4, 2-5 females per replicate), whereas others (n = 19) were housed in larger groups (each consisting of 16 individuals, n replicates = 3, 1-5 subjects per replicate). As part of our analyses, we investigated whether subjects that were housed in smaller groups as chicks differed in their performance in any test compared to subjects housed in larger groups. We also investigated whether there were any differences in test performance between replicates. How these analyses were conducted, and the results we obtained from them, are detailed below.

Analyses

To investigate whether housing as chicks affected our subjects' performance in the detour test, foraging test or simulated predator attack, used in the current study, we used Mann-Whitney-U tests. To explore whether housing as chicks influenced whether subjects won or lost contests as adults, in the current study, we used Fisher's exact tests. For this latter investigation, we used only data from contests in which subjects either won, or lost, all contests against opponents that were housed differently from them (seven subjects that were housed in smaller groups and seven that were housed in larger groups fulfilled this criterion). If we found an effect of how subjects were housed as chicks on any of our measures, for these measures, we performed separate analyses on data from subjects housed in smaller groups, and subjects housed in larger groups, as chicks. To investigate whether the particular replicate subjects had been housed in, as chicks, affected their performance in tests, we first used Kruskal Wallis tests to determine if any differences occurred and then, if differences were detected, Dunn Tests to determine which replicates were significantly different. All analyses were conducted in R studio (v. 3.5.2).

Results

How subjects were housed as chicks did not affect impulsive action or persistence measured in a detour test. However, subjects that were housed in smaller groups as chicks were less persistent in our foraging test, that is showed lower 'Persistence FT' (subjects housed in smaller groups as chicks: mean ± SE = 8.10 ± 0.81; subjects housed in larger groups as chicks: mean ± SE = 11.82 ± 1.39, w = 37, p = 0.02). In addition, subjects that were housed in larger groups as chicks were quicker to return to foraging after the simulated predator attack, even if they appeared to find this attack threatening (i.e., showed higher levels of 'Risk taking' during this test) compared to subjects that were housed in smaller groups as chicks (subjects housed in smaller groups as chicks: mean ± SE = 9.51 ± 2.01; subjects housed in larger groups as chicks: mean ± SE = 22.58 ± 4.26, w = 43, p = 0.04). No other behavioural measures were affected by how subjects were housed as chicks (w = 51 – 102, p > 0.09). Further, whether subjects were housed in smaller or larger groups as chicks did not affect how likely they were to win contests in this study (Fisher's exact test: odds ratio = 0.56, p = 1, 95% confidence interval = 0.03, 7.71). Therefore, for analyses involving 'Persistence FT' or 'Risk taking' subjects that were housed in smaller, and subjects that were housed in larger, groups as chicks were considered separately. For all other analyses, all subjects were considered together. We only found an effect of what replicate subjects belonged to, as chicks, in 'Persistence FT' (Kruskal-Wallis chi-squared = 13.43, p = 0.04). However, as there was only one significant pairwise comparison in 'Persistence FT' between the different replicates, what replicate subjects belonged to, as chicks, was not considered in further analyses.
